# Individual Cognitive Social Capital and Its Relationship with Pain and Sick Leave Due to Pain in the Austrian Population

**DOI:** 10.1371/journal.pone.0157909

**Published:** 2016-06-20

**Authors:** Johanna Muckenhuber, Lorenz Pollak, Katharina Viktoria Stein, Thomas Ernst Dorner

**Affiliations:** 1 Department of Sociology, University Graz, Graz, Austria; 2 Institute of Social Medicine, Centre for Public Health, Medical University Vienna, Vienna, Austria; Iranian Institute for Health Sciences Research, ACECR, ISLAMIC REPUBLIC OF IRAN

## Abstract

**Background:**

Individual cognitive social capital has repeatedly been shown to be linked to health disparities in many dimensions. The aim of the study was to investigate the association between social capital and pain-related measures.

**Methods:**

15,474 subjects older than 15 years were personally interviewed on subjective health, quality of life, health behaviour, and utilisation of healthcare in the Austrian Health Interview Survey. An indicator for cognitive social capital at the individual level consisting of nine questions targeted at different social resources was built and its association with pain-related items analysed.

**Results:**

Odds ratios, adjusted for age, chronic diseases, and educational level for having suffered from severe pain in the last 12 months were 2.02 (95% CI 1.77–2.03) in the lowest tertile and 1.30 (95% CI 1.14–1.47) in the middle tertile of social capital for men. The corresponding odds ratios for women were 2.28 (95% CI 2.01–2.59) and 1.30 (95% CI 1.15–1.46). In both sexes, pain intensity increased significantly with decreasing level of social capital. The proportion of subjects that have been on sick leave in the last 12 months due to pain were 16.3%, 12.0%, and 7.7% (P<0.001) from lowest to highest tertile of social capital in men, and 16.5%, 12.3%, and 6.7%, respectively (P<0.001) in women.

**Conclusion:**

Our findings indicate that low cognitive social capital at individual level is significantly associated not only with higher prevalence of pain and higher pain intensity, but also with a higher chance for sick leave due to pain in employed subjects.

## Background

There is repeated and recent evidence that social capital is linked to disparities in health outcomes: in a literature review from Kim et al. social cohesion as one dimension of social capital was consistently associated with lower overall mortality and better physical health [1]. These results were most marked at the individual (micro) level and in countries with high levels of economic and social inequality and weak safety nets, like the United States of America [2]. Recently, it has been shown that even in more egalitarian societies like Sweden, low general social trust and low social capital among adolescents is linked to higher levels of depression, psychosomatic symptoms and musculoskeletal pain [3].

### Many definitions of social capital

A certain vagueness about the term “social capital” [4] can be attributed to its varying definitions over the last decades from different scientific disciplines: sociology [5], political science [6], economics [7], psychology [8] and public health [9].

The French sociologist Pierre Bourdieu defined social capital as “the aggregate of actual or potential resources linked to possession of a durable network”[5]. Bourdieu and subsequent proponents of his theories focused on specific social resources that are inherent in an individual’s social network. Their concept of social capital has been called the “*social network*”school of social capital [9].

In contrast to Bourdieu’s ideas there is the “*social cohesion*”school of social capital which emphasizes interpersonal trust, associational membership and norms of reciprocity as the core concepts of social capital [6].

#### Cognitive social capital

Another important division of social capital is between *cognitive* and *structural* aspects: Cognitive social capital refers to an individual’s subjective perceptions of accessible social resources, whereas structural social capital refers to actual social activities in formal or informal networks (civic engagement and social participation) that can be objectively measured [10].

#### Pathways of social capital affecting health

There are several postulated mechanisms of how social capital could affect health at the individual level: the three main hypothesised pathways are *social influence* (regulation of health related behaviours by other members of the same social group), *social engagement* (commitment to shared behaviours that affect health outcomes) and the *exchange of social support* (providing interpersonal and emotional resources to improve coping strategies with stressors) [8]. Reverse causation is another possible explanation. It is also possible that bad health could affect social capital at an individual level.

#### Social capital and pain

Pain is a leading reason for primary care consultations and plays a dominant role in health care [11,12], representing a major concern for health care systems and for the society in general [13]. Pain is one of the most common causes for lost productive time, reduced work performance and sick leave [14]. It has been shown in several studies that chronic pain conditions are associated with poor quality of life [15–18].

To understand the whole impact of pain on an individual’s life it is helpful to take into account not only the biomedical dimension, but also psychological and social factors, as suggested in the *biopsychosocial* model [19]. There is evidence that biological factors prevail at the onset of pain, but psychological and social factors (e.g. occupational status, marital status, anxiety, depression) are central aspects in the process of developing chronic pain [20,21].

Social support, which can be regarded as one form of social capital [22], has been shown to be helpful in coping with chronic pain [23,24], whereas lack of social support favours the development of pain [25]. Several studies found poor mental health status and psychological conditions in people with low socio-economic status, in particular depression, to be causal factors for increased extent of pain and diverging pain perception [26,27].

The association between socio-economic status and pain has been thoroughly investigated: there is strong evidence that low levels of education, income or occupational status correlate with increased prevalence of pain, augmented intensity of pain and increased number of painful body areas [17,28]. Socio-economic status plays a major role in determining an individual’s social capital [29,30]. On the other hand, social capital *per se* refers to a broader concept: it encompasses an individual’s entire social network and all its embedded and accessible social resources. However, scientific literature on the association of social capital and pain is scarce.

In accordance with the biopsychosocial model we hypothesised that there should be a strong link between low individual social capital and higher levels of pain. A confirmation of this theory would represent a basis for further pain-related trials with emphasis on social interventions. Such clinical trials could consequently lead to new approaches in chronic pain management.

## Methods

### Survey design

This study was approved by the Ethics Committee of the Medical University Vienna (EC # 770/2011). The database of this study is a questionnaire. All participants agreed to participate to the study but no additional informed consent was given. No additional patient records or information was used. The data of the questionnaire used for our study was completely anonymized and de-identified prior to analysis.

The database used for our analysis was the Austrian Health Interview Survey (AT-HIS) 2006–07 [31]. The survey was commissioned by the Austrian Federal Ministry of Health, Family and Youth and was carried out by Statistics Austria, the national statistics agency. This survey represents a micro-census of a representative sample of the entire Austrian population, with the aim to gain knowledge about subjective health, quality of life, health behaviour and utilization of the healthcare system. To achieve a maximum of representativeness the sample was stratified by geographic region, with the same number of subjects being included from each region. The subjects were interviewed between March 2006 and March 2007 by trained interviewers using CAPI (computer assisted personal interviewing). The gross sample size was 25,130 people, older than 15 years. The response rate was 63.1%. The questionnaire was designed based on the European Core Health Interview Survey (EC-HIS) and has been adapted for Austria by an expert panel. In order to account for the stratification of the sample the data were weighted by geographic region, age, and sex. Missing values were systematised in order to differentiate between different kinds of missing values.

#### Pain related survey items

Regarding pain, the subjects were asked “Did you suffer from severe pain in one or more than one body site during the last 12 months?”If this was answered with “yes”the responders were shown a picture of the body, with 14 different body sites and asked to identify the region or regions, in which they experienced the pain. For each region with pain the subjects were asked if they had been on sick leave because of the pain in the last 12 months. The proceeding question was, whether the pain in that region had also occurred during the last seven days. If this was the case, the subjects were asked to rate the intensity of pain with the help of a visual analogue scale, 1 denominating “very little pain” and 10 “heaviest pain imaginable”. Sick leave analysis was performed only in subjects who indicated that they were currently employed. Thus, retired or unemployed subjects, students, subjects in early retirement or disability pension, subjects in military or civilian service and subjects on maternity or paternity leave were excluded from these analyses.

#### Social capital indicator and corresponding survey items

Giving consideration to the ongoing scientific debate on the concepts for social capital and its measurement, the present study utilised Bourdieu’s concept of social capital: we presumed the exploration of various resources that are embedded in an individual’s social network to be of most clinical significance for day-to-day work with patients.

We focused on individuals’ subjective perceptions of accessible social resources, corresponding to “cognitive social capital” [10]. Our motivation for concentrating on cognitive aspects was that high levels of cognitive social capital are linked to good mental health [32], which in turn seems to be a protective factor for developing chronic pain conditions [27]. Moreover, pain and cognitive social capital share one common ground: they are both completely based on a patient’s self-perception and subjective experience.

Therefore, we constructed an indicator by means of a summarised scale, using nine items from the WHOQOL-Bref inventory [33]. In accordance with Bourdieu’s concept of social capital [5] and with previous research [34] we selected the following items: “How widely available is the information that you need in your day-to-day life?”; “To what extent do you have opportunity for leisure activities?”; “How safe do you feel in your daily life?”; “How satisfied are you with your transport?”; “How satisfied are you with your access to health services?”; “How satisfied are you with your personal relationships?”; “How satisfied are you with the support you get from your friends?”; “How satisfied are you with the conditions at your place of residence”

The constructed indicator for cognitive social capital reached a Cronbach’s Alpha of 0.792 and included values for the nine questions corresponding to the items mentioned above. Each response was rated on a 5-point Likert scale. The resulting indicator was computed by summarising the values for those questions, ranging from 9 to 45 points in total. Descriptive analyses indicated a non-linear relationship between social capital and sick leave. Therefore, and in order to compare groups, the indicator was recoded into a new variable establishing tertiles of individuals with low, moderate, and high social capital. The lowest tertile ranged from 9 to 35 points, the medium tertile from 36 to 39 points and the highest tertile from 40 to 45 points.

### Statistical evaluation

As control variables in the regression models age was used in steps of five years, and level of education was measured as ordinal variable with three levels of education (primary, secondary, and tertiary education). The existence of chronic diseases in the respondents was asked with the question “Do you have a chronic, that means long-term, disease or a chronic health problem?” The analysis was done separately for men and women in order to investigate if there are gender differences.

Bivariate analyses were undertaken by means of cross-tabs, significance was tested with Chi²-test. For metric variables the mean was computed and univariate analysis of covariance (oneway ANOVA in SPSS) was applied. Binary logistic regression models were calculated with having suffered from pain and having been on sick leave due to pain as the dependent variables, respectively. Social capital, highest education level, and presence of chronic disease were used as categorical and age as metric independent variables in the logistic regression analyses. The results of all logistic regression models are presented as odds ratios with 95% confidence intervals. Nagelkerkes’ R² is presented as a measure of model-fit. All results were stratified by sex. Calculations were done using SPSS 20.

## Results

Descriptive analyses showed that 35.4% of men and 41.5% of women (P<0.001) were suffering from severe pain in the last 12 months. Regarding a shorter time span 22.7% of men and 30.3% of women (P<0.001) experienced severe pain in the last seven days before questioning. Pain is also an issue for sick leave, 11.2% of men and 10.9% of women (P<0.680) of all currently employed subjects were on sick leave due to pain in the last 12 months. When confining the sample to employed subjects who declared having suffered from severe pain in the last 12 months the proportion of those who were on sick leave due to pain was 35.6% in men and 29.5% in women, respectively (P = 0.001).

Subjects with lower social capital had a higher chance of severe pain. In unadjusted analyses, the prevalence of severe pain was 51.0% in male subjects in the lowest tertile of social capital, 33.4% in the medium and 24.4% in the tertile with the highest values for social capital (P<0.001). In women, the prevalence of severe pain was 58.6%, 38.0% and 29.4% in subjects with the lowest to the highest tertile of social capital, respectively. Even after adjustment for age, education, and presence of a chronic disease, the chance of suffering from severe pain increased with decreasing social capital (Table 1). The model-fit statistics (Nagelkerkes R²) is not very high (Table 1). That shows that even though we can see that social capital is an important factor for the prevalence of severe pain and even though we have included age, chronic diseases and educational level into the model, apparently there are other important factors that are associated to the prevalence of severe pain.

**Table 1:** Association between social capital and prevalence of severe pain within the last 12 months, results of a logistic regression model.

	Men				Women			
	N	OR*	95% CI	P	N	OR*	95% CI	P
Social capital in lowest tertile	2114	2.02	1.77–2.30	<0.001	2558	2.28	2.01–2.59	<0.001
Social capital in middle tertile	2548	1.30	1.14–1.47	<0.001	2648	1.30	1.15–1.46	<0.001
Social capital in highest tertile	2790	1			2814	1		
Nagelkerkes R²	0.191				0.219			

*adjusted for age, chronic diseases, and educational level

Similarly, unadjusted analyses show that employed subjects with lower social capital had a higher rate of having been on sick leave due to pain. In men, the proportion of subjects having been on sick leave in the last 12 months due to pain was 16.3%, 12.0%, and 7.7% (P<0.001) in the lowest to highest tertiles of social capital. The corresponding values for women were 16.5%, 12.3%, and 6.7% (P<0.011).

When analysing a different subsample, just the group of employed subjects who were suffering from severe pain in the last 12 months a slightly different and non-linear pattern can be observed. In this subsample the proportion of those who were on sick leave due to pain was 36.0%, 39.8%, and 30.9% (P = 0.015) in men with lowest to highest tertiles for social capital, and 31.5%, 33.3%, and 23.3% (P = 0.002), respectively for women. After adjustment for age, education level and chronic disease, women in the lowest and middle tertile of social capital had a significantly higher chance of being on sick leave due to pain compared to those in the highest tertile (Table 2). In men these values were significant in the middle tertile of social capital when compared to the highest. Regarding the low values of the model fit (Table 2) we can state that apparently there are other very important factors that have a strong influence on sickness absence due to pain.

**Table 2:** Association between social capital and the chance of having been on sick leave due to pain in currently employed subjects who were suffering from severe pain within the last 12 months, results of a logistic regression model.

	Men				Women			
	N	OR*	95% CI	P	N	OR*	95% CI	P
Social capital in lowest tertile	469	1.18	0.89–1.57	0.248	403	1.41	1.02–1.93	0.036
Social capital in middle tertile	493	1.41	1.08–1.85	0.013	471	1.61	1.19–2.16	0.002
Social capital in highest tertile	479	1			430	1		
Nagelkerkes R²	0.025				0.033			

*adjusted for age, chronic diseases, and educational level

In subjects who were suffering from severe pain in the last 7 days, pain severity was 5.66 (95% CI 5.57–5.76) in men and 5.93 (95% CI 5.84–6.01) in women, as reported on the 10-part VAS. In both sexes, pain intensity increased with decreasing level of social capital (Table 3).

**Table 3:** Intensity of pain (mean values on a 10-step VAS) in subjects suffering from severe pain within the last 7 days by social capital.

	Men			Women		
	N	Mean	95% CI	N	Mean	95% CI
Social capital in lowest tertile	808	6.2	6.1–6.4	1238	6.3	6.2–6.5
Social capital in middle tertile	534	5.3	5.2–5.5	702	5.6	5.4–5.7
Social capital in highest tertile	352	4.9	4.7–5.1	488	5.3	5.2–5.5
P (Oneway ANOVA)	<0.001			<0.001		

## Discussion

### Main Findings

We found a strong association between social capital as measured by an individual level cognitive social capital indicator and prevalence of severe pain, independent of potential confounding factors like age, education or chronic disease: decreasing social capital was significantly linked to increasing prevalence of pain and increasing intensity of recent pain. Women had even higher chances of suffering from pain when compared to men in the same tertiles of social capital. Interestingly, gender differences became less pronounced after adjustment for covariates in our regression model. Higher social capital was significantly associated with lower overall percentage of sick leave due to pain in both sexes.

We consider these results to be in accordance with the biopsychosocial concept of pain [20,35], notably with its psychological and social components and their inherent subjective nature. Thus, the prevalence of pain and the perception of pain are strongly connected to an individual’s subjective experience of pain. It has been shown that the perceived disability due to pain depends on various social factors [17,27]. Similarly, our findings support the theory that social capital affects the process of pain development and the perception of pain. Most likely mechanisms include social influence, social engagement, exchange of social support [8] and influences on coping strategies [23]. This could be one possible explanation for the associations found in our study between individual cognitive social capital and pain.

However, when taking into account just those employed subjects who declared having suffered from severe pain during the course of the last year we found the most pronounced associations with sick leave rates in the middle tertiles of social capital. These proportions did not change after adjusting for age, education and chronic disease. It has to be noted that sick leave rates in men with severe pain did not differ significantly between the lowest and the highest tertile of social capital.

Thus, sick leave rates due to chronic and severe pain conditions did not show a linear association between social capital and sick leave. After adjustment for confounding factors, highest sick leave rates were still at the middle tertiles of social capital in both sexes. This is a somewhat unexpected result. We argue that this result can be explained by the distribution of social capital. We found that social capital is significantly associated to the prevalence of severe pain. Therefore, we know, that social capital is differently distributed between individuals who declare to have suffered from severe pain during the last 12 months and those who didn't. Therefore, it is possible that there is a linear pattern between the distribution of social capital and percentages of sickness absence due to pain in the one subsample and a non-linear pattern in the other one.

In general, we can state that in both cases—for the subsample of all employees and for the subsample of employees who suffered from pain during the last 12 months—individuals with high social capital have the lowest risk for sick leave due to pain when compared to persons with middle social capital and when compared to persons with low social capital.

But there is a difference in the pattern of the association. We find a linear pattern for the broader sample of all employees but the association is not linear for the case of employees who suffered from severe pain during the last 12 months.

We found that persons with middle social capital have a higher risk for sick leave due to pain when compared to the risk of persons with high social capital. In contrast men with low social capital don’t have a significantly increased risk for sick leave due to pain when compared to men with high social capital. For women we find, that the risk for sick leave due to pain of women with low social capital is slightly higher than the risk of women with high social capital, but the effect seems to be stronger for the comparison between middle and high social capital.

There are different possible explanations for the finding of a linear pattern that more persons with low than with high social capital report that they have been on sick leave due to pain.

As a first point we should mention that it is very likely that persons with lower social capital experience more and more often acute pain than persons with high social capital. This explanation is supported by our finding that persons with low social capital report higher intensity of pain than persons with high social capital do. If they experience more often acute pain it is comprehensible that they report more sick leave.

But apart from this we can also state that in general individuals with low social capital are employed in job where they have just very little work adjustment latitude which means that they just have little possibilities to adjust their working day according to their physical abilities. When we think of a construction worker for example it is very likely that he goes on sick leave because of pain because it will be pretty much more difficult and even more painful and for him to work when compared to an employee in an office.

But how can we explain the finding of a non-linear pattern in the subsample of individuals how report that they have suffered from pain during the last 12 months. We assume similar explanations for the finding that more individuals with middle than with high social capital report that they were on sick leave due to pain and that more individuals with low than with high social capital report sick leave. While we found in the sample of all employees that more persons with low than with middle social capital report sick leave, for the subsample of employees who experienced severe pain during the last 12 months we find very small differences between these two groups. We even found that slightly more persons with middle than with low social capital report sick leave. We argue, that persons who experienced severe and perhaps persisting pain during the last 12 months and who in addition have a very low social capital are especially vulnerable. Persons with low social capital often have jobs where their performance is weakened by persisting pain (like back ache) therefore it is likely that they stand under pressure and that they are in particular afraid to lose their jobs. As a consequence, it is very likely that they don’t go easily on sick leave because of acute pain that they experience. Individuals with middle social capital in contrast who experienced severe pain are also vulnerable but they might experience more job security, that’s why they can take the risk to go on sick leave because of acute pain without being afraid to lose their job

As expected and shown in previous studies [24], the proportion of women suffering from severe pain in the course of the last seven days or the last twelve months was significantly higher than the respective proportion of men. On the other hand, men with severe pain conditions were more likely to be on sick leave due to pain during the last year than women. Thus, although there is evidence that pain in general is more common in women, men apparently tend to be more likely to be on sick leave when suffering from chronic and severe pain.

### Strengths and limitations

The findings of the present study are based on a national health survey with optimal representative data for the general Austrian population. Thank you for this comment we have expanded our argument at this point: However, it has to be stressed that a response rate of 61.3% could generate a possible selection bias because it might be the case that there is a difference in the response patterns of the 61.3% and the 38.7% who didn’t respond. But nevertheless we can state that a response rate of 61.3% corresponds to common response rates in social surveys like this one.

We took care that our measurement tools comprised different facets of their underlying concepts: our social capital indicator consisted of nine different types of social resources from various areas of daily life. Cronbach’s Alpha was provided as reliability assessment via internal consistency. Regarding pain evaluation, we incorporated three different measures: 1-year period prevalence of severe pain, pain intensity, and sick leave rates due to pain.

To our knowledge this is the first study that examined associations between social capital and sick leave due to pain. Therefore, it represents an important contribution to the existing literature on social capital and its influences on health outcomes.

Our approach of investigating social capital has to be regarded under the following limitations: We took only individual (micro) level measures into account but did not evaluate social capital at the (meso) neighbourhood level or at even higher (macro) levels like districts or state regions. Hence a multi-level approach as favoured by some social capital researchers [36] was not applicable to our social capital indicator.

Another common distinction is drawn between *bonding* and *bridging* social capital [37]. Bonding social capital refers to accessible social resources from other group members with similar features like class, race or social status. Bridging social capital describes resources that can be accessed across social boundaries thus connecting different social groups. Regrettably, data available from the AT-HIS was not sufficient to differentiate between bridging and bonding social capital.

Regarding the actual measurement of individual social capital we focused on concrete *social resources* (e.g. feeling of safety, social support, access to vital information). Other types of measures like *social network structure*, *total amount of social resources* or *diversity of social resources* were not part of the survey. As stated above, we confined our social capital indicator to cognitive aspects, whereas other authors prefer structural social capital factors, like documented participation in neighbourhood associations or civic organizations, to describe the level of social capital [38].

It has to be clarified that the original survey was not designed to measure social capital in the first place. The selected survey items originated from the well known quality-of-life assessment tool WHOQOL-Bref [33]. However, social capital evaluation clearly overlaps QoL assessment in certain areas: in analysing survey items that were related to social capital we were able to gain new insights into its relationship with pain and sick leave rates.

Lastly, given the fact that the present study is cross-sectional, we cannot infer from our study results that low social capital is a causal factor for developing more intense or more frequent pain conditions. It might as well be, that pain and in particular frequent sick leave affects social capital at an individual level. So we can state, that reverse causation is another possible explanation.

To investigate the causality in the proven association between social capital and pain further longitudinal and prospective studies are needed.

## Conclusion

The results of the present study show that low levels of individual cognitive social capital are significantly associated with higher prevalence of pain and higher intensity of pain. For employees we found that high social capital is significantly associated to lower rates of sick leave. But there are more complex patterns of association regarding middle and low social capital and sick leave rates.

Our findings could represent a basis for further clinical trials that investigate the impact of social capital interventions on pain-related health outcomes (e.g. sick leave rates). It has been shown that chronic pain patients prefer a comprehensive disease-centred conversation with their physician over other pain-reducing interventions [18,28]. Thus, we believe that exploration and facilitation of social capital could take place during a treatment conversation. The effectiveness of such measures remains to be investigated.

Unfortunately, there is no consensus about the nature of interventions needed to build social capital [9]. At the community level, social capital can be arguably improved by encouraging the formation of civic associations and by promoting management and leadership skills of community members [39]. Scientific literature on building social capital at the individual level is particularly sparse.

Taking our study results into account, one can however underline the importance of the topic and advocate for more public health measures targeting patients with low social capital. Health literacy would be such a multi-level approach, and its values have already extensively been described in the literature [39].

### Conflicts of interest

None declared.

**Key points**

- Low levels of individual social capital are strongly associated with increased prevalence of pain and intensity of pain. High social capital is significantly associated to lower rates of sick leave. The pattern of association for middle and low social capital and sick leave rates is more complex.
- This is the first study to examine associations between social capital and sick leave due to pain.
- We hypothesize that public health measures targeting patients with low individual cognitive social capital could improve pain-related health outcomes and sick leave rates due to pain
